# Liver Involvement in Patients with Rare *MBOAT7* Variants and Intellectual Disability: A Case Report and Literature Review

**DOI:** 10.3390/genes14081633

**Published:** 2023-08-16

**Authors:** Luisa Ronzoni, Matteo Mureddu, Francesco Malvestiti, Vittoria Moretti, Cristiana Bianco, Giulia Periti, Margherita Baldassarri, Francesca Ariani, Anna Carrer, Serena Pelusi, Alessandra Renieri, Daniele Prati, Luca Valenti

**Affiliations:** 1 Biological Resource Center, and Department of Transfusion Medicine, Fondazione IRCCS Ca’ Granda Ospedale Maggiore Policlinico Milano, 20122 Milan, Italy; 2 Department of Pathophysiology and Transplantation, Università degli Studi di Milano, 20122 Milan, Italy; 3 Medical Genetics, University of Siena, 53100 Siena, Italy; 4 Med Biotech Hub and Competence Center, Department of Medical Biotechnologies, University of Siena, 53100 Siena, Italy; 5 Genetica Medica, Azienda Ospedaliero-Universitaria Senese, 53100 Siena, Italy

**Keywords:** membrane-bound O-acyltransferase domain-containing 7 (MBOAT7), LPIAT1, intellectual disability, steatotic liver disease, fatty liver disease

## Abstract

The membrane-bound O-acyltransferase domain-containing 7 (MBOAT7) protein is an acyltransferase catalyzing arachidonic acid incorporation into lysophosphatidylinositol. Patients with rare, biallelic loss-of-function variants of the *MBOAT7* gene display intellectual disability with neurodevelopmental defects. The rs641738 inherited variant associated with reduced hepatic MBOAT7 expression has been linked to steatotic liver disease susceptibility. However, the impact of biallelic loss-of-function *MBOAT7* variants on liver disease is not known. We report on a 2-year-old girl with *MBOAT7*-related intellectual disability and steatotic liver disease, confirming that *MBOAT7* loss-of-function predisposes to liver disease.

## 1. Introduction

Membrane-bound O-acyltransferase domain-containing 7 (MBOAT7) protein, encoded by the *MBOAT7* gene, is a lysophosphatidylinositol acyl-transferase catalyzing phosphatidylinositol (PI) acyl-chain remodeling in the Lands cycle responsible for the selective placement of acyl chains in phospholipids, regulating the asymmetry and properties of cellular membranes. MBOAT7 is a 472-amino-acid-long protein anchored to the endoplasmic reticulum (ER), lipid droplets, and mitochondria-associated membranes, which uniquely synthesizes PI from arachidonoyl-CoA and lyso-PI substrates [1]. N-terminal residues on the ER luminal side have recently been identified to determine phospholipid selectivity [2].

Rare, deleterious biallelic variants in the *MBOAT7* gene have been linked to a brain developmental disorder (OMIM #617188) [3] characterized by intellectual disability (ID), autism spectrum disorders (ASDs), early-onset seizures that are usually well controlled by common anti-epileptic drugs, speech impairment, abnormal motor coordination, and brain malformations [4,5,6,7,8,9,10,11]. The mechanism by which *MBOAT7* variants cause neurological diseases is not yet well understood, but experimental models suggest that it involves altered myelination due to the impaired metabolism of phospholipids [3,12].

Notably, the common inherited rs641738C>T variant at the *MBOAT7-TMC4* locus has also been linked to an increased risk of steatotic liver disease (SLD) [13]. The rs641738C>T variant is associated with the increased development and severity of the entire steatotic liver disease spectrum, from steatosis to hepatocellular carcinoma (HCC) in individuals of European descent [14,15,16]. The association between rs641738 and steatosis has been hypothesized to be accounted for by the downregulation of MBOAT7 hepatic protein expression, resulting in changes in hepatic PI-acyl-chain remodeling and increased lipogenesis [17,18,19]. The rs641738 variant is not likely the causal variant predisposing to non-alcoholic fatty liver disease (NAFLD) and HCC, but other variants in the 3′UTR region of *MBOAT7*, in linkage with rs641738, may be involved in the regulation of *MBOAT7* mRNA stability [14]. However, the exact mechanism by which *MBOAT7* rs641738 predisposes to steatosis is still disputed [20]. Moreover, the impact of rare deleterious *MBOAT7* variants on liver function has not yet been assessed.

Herein, we describe the case of a 2-year-old Italian girl with a rare homozygous loss-of-function *MBOAT7* variant, neurodevelopmental delay, and steatotic liver disease.

## 2. Case Report and Genetic Analysis

The patient, a 2-year-old Italian girl, was referred for a consultation at the Fondazione due to a recent diagnosis of *MBOAT7* biallelic mutations.

She is the first child of unrelated, healthy, Italian parents, whose families originated from a small city in Tuscany. She was born at term after an uneventful pregnancy; her auxological parameters at birth were normal, with an APGAR score of 9 at 1 min and 10 at 5 min. At 2 months of age, she presented with epileptic seizures during sleep. Electroencephalography (EEG) showed multifocal paroxysmal activity, prevalent in the right hemisphere. Magnetic resonance imaging (MRI) showed moderate dilatation of the ventricular system and small thinning of the cerebral cortex. A global developmental delay was evident from the age of 7 months, with no control of the trunk, poor eye contact, and intermittent stereotypes involving both hands. MRI was repeated at 1 year of age; it highlighted the bilateral absence of opercularization and a delay in the myelinization of the frontal and parietal lobes.

**Figure 1:**
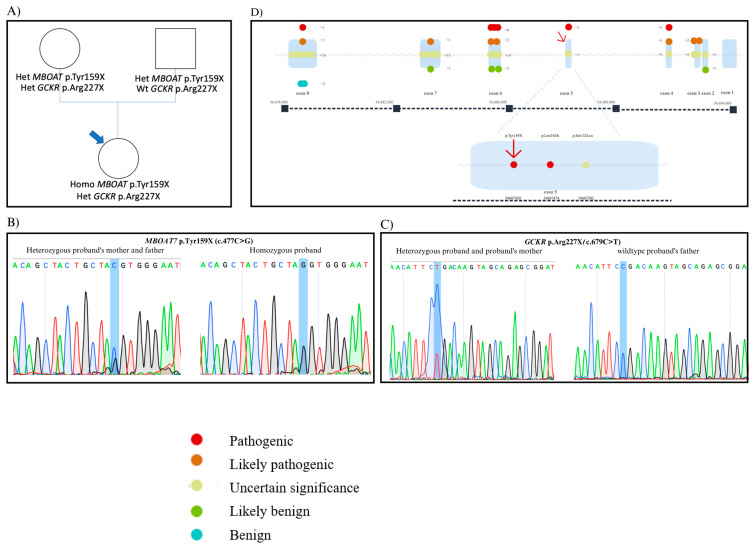
(**A**): Family tree of the proband. (**B**): Results of Sanger sequencing of MBOAT7 exon 5 in the proband and their carrier parents. (**C**): Results of Sanger sequencing of GCKR exon 9 in the proband and their carrier parents. (**D**): Schematic illustration of the MBOAT7 protein structure with the previously identified variants classified as pathogenic, likely pathogenic, uncertain significance, likely benign, and benign. Zoom imaging of MBOAT7 exon 5 with the variant described in this study (indicated by the red arrow) and the other known variants (modified from the ClinVar Website; reference genome: GRCh37). Abbreviations: Homo: homozygous; Het: heterozygous; Wt: wild-type.

At 1 year of age, *MBOAT7*-related development delay disorder was diagnosed due to the presence of a rare, homozygous, truncating variant in the *MBOAT7* gene (NM_024298: c.477C>G; p.Tyr159X), most likely resulting in the functional knockout of MBOAT7. Both of the parents were heterozygous for the same variant (Figure 1A,B).

The patient underwent regular clinical neurological follow-up and was treated with phenobarbital (8 mg/kg/die) and then substituted with valproic acid (32 mg/kg/die), with good control of the seizures. Her valproate circulating levels, periodically evaluated, were within the normal range.

**Figure 2:**
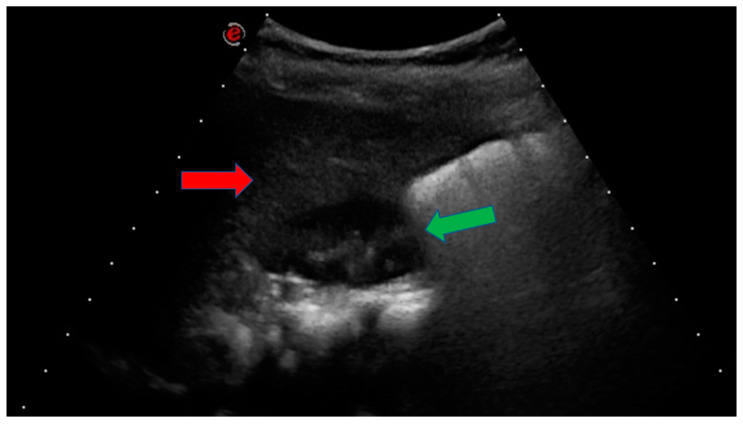
The proband’s abdominal ultrasonography: the liver (red arrow) appears hyperechogenic compared to the kidney (green arrow).

At 2 years of age, when she came to our attention, her aspartate transaminase (AST) levels were increased (42 U/L), with the other liver enzymes in the normal range (Table 1). Her TSH levels were normal, although, in previous examinations, subclinical hypothyroidism was detected, with TSH levels of 8.9 mU/L and fT4 values in the normal range. During the clinical evaluation, her weight was at the 25th centile, her height was at the 67th centile, and her head circumference was at the 50th centile. Abdominal ultrasonography (US) highlighted the presence of liver hyperechogenic areas, consistent with liver steatosis (Figure 2). A liver biopsy was discussed but not performed due to the young age of the proband and her clinical condition.

To better evaluate the possible genetic predisposition to liver diseases, we performed an analysis of a panel of genes related to hepatic and metabolic alterations.

After informed written consent was obtained from the parents, a peripheral blood sample was collected. DNA was extracted and next-generation sequencing (NGS) of a panel of 82 liver-related genes was performed, as previously described [21].

Besides confirming the homozygosity for the truncating *MBOAT7* variant, a rare heterozygous nonsense variant was identified in the glucokinase receptor (*GCKR*) gene (NM_001486: c.679C>T; p.Arg227X; rs149847328), which is predicted to predispose to steatosis by promoting hepatic lipogenesis [22]. Sanger sequencing validated the results of the NGS, and the segregation analysis revealed the maternal origin of such a variant (Figure 1C).

The polygenic risk score for steatotic liver disease (PRS-5), based on the evaluation of common genetic variants in *PNPLA3* (rs738409), *TM6SF2* (rs58542926), *GCKR* (rs1260326), *MBOAT7* (rs641738), and *HSD17B13* (rs72613567) genes [23,24], was in the normal range (Table 1). However, it should be noted that rare variants in *MBOAT7* and *GCKR* were not considered for PRS-5 determination.

Considering the young age of the proband, the evidence of steatosis with abnormal AST levels, and the possible effects of the two rare variants on steatosis progression, it was recommended to maintain a regular hepatological follow-up, including vibration-controlled transient elastography (FibroScan) with an appropriate pediatric probe, to predict the severity of liver disease. Moreover, a diet rich in PI and arachidonic acid was suggested.

## 3. Discussion

We reported the case of a 2-year-old girl with a rare, homozygous, severe loss-of-function *MBOAT7* variant suffering from epilepsy and neurodevelopmental impairment. She shared the same clinical features previously described in the literature of patients with biallelic loss-of-function *MBOAT7* variants (Table 2). Specifically, she experienced developmental delay with poor motor coordination, speech delay, and focal seizures treated with antiepileptic drugs. Noteworthy, abdominal ultrasound evaluation revealed the presence of hepatic steatosis, despite the patient’s very young age.

In the previously described cases with *MBOAT*-related ID, liver involvement was reported. Only in three cases from two families was liver function assessed, and no signs of steatotic liver diseases were recorded [7]. Different causes could explain these discrepancies [7]: the majority of the reported patients are in their childhood, and liver disease might manifest later in life; ultrasonography could have low sensitivity in detecting fatty liver, and steatosis not affecting liver function tests could have gone unnoticed; and finally, carriers of *MBOAT7* variants had low adiposity, which might have camouflaged the absence of *MBOAT7* product in the liver.

Steatotic liver disease has been linked to the downregulation of MBOAT7 liver expression, both in human studies with in vitro cell cultures and in vivo mouse models. In vivo and in vitro studies suggest that MBOAT7 deficiency leads to hepatocellular lipid accumulation through de novo lipogenesis mediated by sterol regulatory element–binding protein-1 (SREBP-1) or non-canonical pathways, resulting in triglycerides synthesis. In fact, the impaired generation of arachidonoyl-PI, consistent with a reduction in MBOAT7 enzymatic activity, leads to the conversion of saturated lyso-PI to triglycerides and enhanced lipogenesis [17,18,25].

In the reported case, the homozygous variant c.477 C>G is located in exon 5 of the *MBOAT7* gene (Figure 1D), and it is predicted to determine the insertion of a premature stop codon at amino acid position 159. The insertion of the premature stop codon (p.Tyr159X) resulted in the generation of an inactive truncated protein, lacking the catalytic pocket formed by residues His356 and Asn321 or even in a premature decay of the *MBOAT7* mRNA transcript with no MBOAT7 production, determining the neurological phenotype. The presence of steatosis, which is very uncommon at her age despite the absence of obesity, is therefore consistent with the notion that the absence of MBOAT7 activity is also a risk factor for liver disease.

However, we cannot rule out that in the present case, steatosis might have also been triggered by the heterozygous variant in the *GCKR* gene. GCKR is a regulatory protein that inhibits glucokinase in the liver and pancreatic cells. The *GCKR* variant p.Arg227X (rs149847328) determines the insertion of a stop codon at position 227, resulting in a non-functional protein. Notably, the aforementioned variant has previously been reported in association with progressive steatotic liver disease, which manifests, however, after diabetes development during middle age [22]. Of note, the mother of our proband carried the same heterozygous variant but did not develop steatosis. On the contrary, our proband developed steatosis at a very young age, and this may be due to the presence of both the homozygous p.Tyr159X MBOAT7 variant and the heterozygous p.Arg227X GCKR variant. It could therefore be speculated that in the mother, haploinsufficiency for the *GCKR* variants is not sufficient to induce steatosis development even in the presence of the monoallelic *MBOAT7* loss-of-function mutation, but it could trigger steatotic liver disease along with biallelic *MBOAT7* loss-of-function mutations and environmental factors, such as valproate, in the proband. 

Indeed, we cannot rule out that therapy with valproate may have contributed to steatosis in this specific patient. Indeed, one of the most frequent and severe adverse side effects of valproate therapy is the development of hepatic steatosis [26]. The mechanisms underlying the development of this liver injury are not fully understood. Recent in vivo and in vitro studies have demonstrated that lipid accumulation caused by valproate treatment could be mediated by increased expression of the pregnane X receptor (PXR)—fatty acid binding protein 4 (FABP4) pathway, while SREBP-1 mediated lipogenesis, accounting for lipid accumulation in MBOAT7 deficiency, may not be involved [27,28]. Thus, liver steatosis could be the result of different interacting metabolic pathways, induced by valproate treatment and MBOAT7 deficiency.

Given the role of MBOAT7 deficiency in predisposing to steatotic liver disease, it has been hypothesized that the upregulation of MBOAT7 expression can improve steatosis. Recent studies on mouse models of steatohepatitis, in which hepatic MBOAT7 was selectively overexpressed, have demonstrated an improvement in serum markers of hepatic injury [29]. Of note, early restoration of MBOAT7 expression may represent an actionable strategy to counteract the neurological manifestations of disorders. Whole-body MBOAT7 knockout mice die within the first month of life, with atrophy of the cerebral cortex and hippocampus due to defective cortical lamination. It could be hypothesized that alterations in arachidonic-acid-containing PI, consequent to MBOAT7 deficiency, could account for dysregulations in cortical lamination development, with neuronal degeneration and increased gliosis [12]. A neuronal-targeted viral gene therapy to overcome *MBOAT7* variants in mice models or in vitro cell cultures could contribute to gaining insights into the pathophysiological mechanisms of MBOAT7-related neurological disorders, paving the way for future clinical trials in human patients.

In conclusion, the present report underlines, for the first time, the importance of liver function evaluation in patients with MBOAT7-related neurodevelopmental disorders. Given the increased risk of liver steatosis even at a young age, evaluation of liver function tests and ultrasonography should be included in routine follow-up evaluations. These results are consistent, although not yet conclusive, with the notion that reduced hepatic MBOAT7 activity predisposes to steatotic liver disease.

## Figures and Tables

**Table 1:** Clinical, biochemical, and genetic features of the proband.

	Present Case	Standard References
Age (year)	2	
Auxological Parameters		
Weight (centile)	25	
Height (centile)	67	
Head circumference (centile)	50	
Blood Chemistry Test		
AST (U/L)	**42**	<32
ALT (U/L)	22	<33
GGT (U/L)	8	<40
ALP (U/L)	189	140–365
Albumin (g/dL)	4.8	3.8–5.4
Bilirubin (mg/dL)	0.33	<1.0
Total cholesterol (mg/dL)	116	108–193
Triglycerides (mg/dL)	63	<170
Glucose (mg/dL)	87	<87
Hb (g/dL)	**9.7**	10.5–15.0
TSH (µUi/L)	4.63	0.7–6.0
Genetic data		
*MBOAT7* (c.477C>G; p.Tyr159X)	Homozygous	
*GCKR* (c.679>T; p.Arg227X)	Heterozygous	
PRS-5	0.266	<0.495
Abdominal US	**Liver hyperechogenic areas**	

Abbreviations: BMI: body mass index; AST: aspartate aminotransferase; ALT: alanine aminotransferase; GGT: γ-glutamyl transferase; ALP: alkaline phosphatase; Hb: hemoglobin; TSH: thyroid-stimulating hormone; PRS-5: polygenic risk score considering 5 risk variants; US: ultrasonography. The values out the standard references are highlighted in bold.

**Table 2:** Comparison of the clinical features of the proband with the cases previously reported.

Clinical Characteristic	Present Case	Previously Described Cases [3,4,5,6,7,8,9,10,11] (n = 60)
Sex	F	M = 32; F = 28
Age	2 years	2 months to 22 years
Developmental delay/intellectual disability	Y	56/60 (94%)
Speech delay/impairment	Y	56/60 (94%)
Motor delay/impairment	Y	53/56 (95%)
Poor coordination/ataxic gait	Y	21/47 (37%)
Axial hypotonia	Y	43/45 (96%)
ASDs/hyperactivity	N/A	29/51 (57%)
Seizures	Y	46/55 (84%)
Microcephaly/macrocephaly	N	15/51 (29%)
Strabismus/retinal degeneration/optic atrophy	N/A	10/21 (48%)
Neuroimaging alterations (reported abnormalities)	Y Ventricles enlargement Thin corpus callosum Frontal polymicrogyria Bilateral delay in opercularization	30/48 (63%) Polymicrogyria: 3/48 (6%) Cortical atrophy: 15/48 (31%) Cerebellar dysgenesis: 9/48 (19%) Hyperintensity of globus pallidus and dentate nuclei: 8/48 (17%)
Obesity	N	5/19 (26%)
Metabolic profile	Increased AST levels	Normal in 23 subjects; n.a. for the others
Liver US	Steatosis	Normal in 3 subjects n.a. for the others

Abbreviations: F: female; M: male; Y: yes; N: no; ASDs: autistic spectrum disorders; AST: aspartate aminotransferase; US: ultrasonography; N/A: not applicable; n.a.: not available.

## Data Availability

The data that support the findings of this study are available from the corresponding author upon request.
